# A chemically consistent graph architecture for massive reaction networks applied to solid-electrolyte interphase formation†

**DOI:** 10.1039/d0sc05647b

**Published:** 2021-02-24

**Authors:** Samuel M. Blau, Hetal D. Patel, Evan Walter Clark Spotte-Smith, Xiaowei Xie, Shyam Dwaraknath, Kristin A. Persson

**Affiliations:** Energy Technologies Area, Lawrence Berkeley National Laboratory Berkeley CA 94720 USA; Department of Materials Science and Engineering, University of California Berkeley CA 94720 USA; Materials Science Division, Lawrence Berkeley National Laboratory Berkeley CA 94720 USA; College of Chemistry, University of California Berkeley CA 94720 USA; Molecular Foundry, Lawrence Berkeley National Laboratory Berkeley CA 94720 USA kapersson@lbl.gov

## Abstract

Modeling reactivity with chemical reaction networks could yield fundamental mechanistic understanding that would expedite the development of processes and technologies for energy storage, medicine, catalysis, and more. Thus far, reaction networks have been limited in size by chemically inconsistent graph representations of multi-reactant reactions (*e.g.* A + B → C) that cannot enforce stoichiometric constraints, precluding the use of optimized shortest-path algorithms. Here, we report a chemically consistent graph architecture that overcomes these limitations using a novel multi-reactant representation and iterative cost-solving procedure. Our approach enables the identification of all low-cost pathways to desired products in massive reaction networks containing reactions of any stoichiometry, allowing for the investigation of vastly more complex systems than previously possible. Leveraging our architecture, we construct the first ever electrochemical reaction network from first-principles thermodynamic calculations to describe the formation of the Li-ion solid electrolyte interphase (SEI), which is critical for passivation of the negative electrode. Using this network comprised of nearly 6000 species and 4.5 million reactions, we interrogate the formation of a key SEI component, lithium ethylene dicarbonate. We automatically identify previously proposed mechanisms as well as multiple novel pathways containing counter-intuitive reactions that have not, to our knowledge, been reported in the literature. We envision that our framework and data-driven methodology will facilitate efforts to engineer the composition-related properties of the SEI – or of any complex chemical process – through selective control of reactivity.

## Abstract 2

A chemically consistent graph architecture enables autonomous identification of novel solid-electrolyte interphase formation pathways from a massive reaction network.

## Graphical abstract

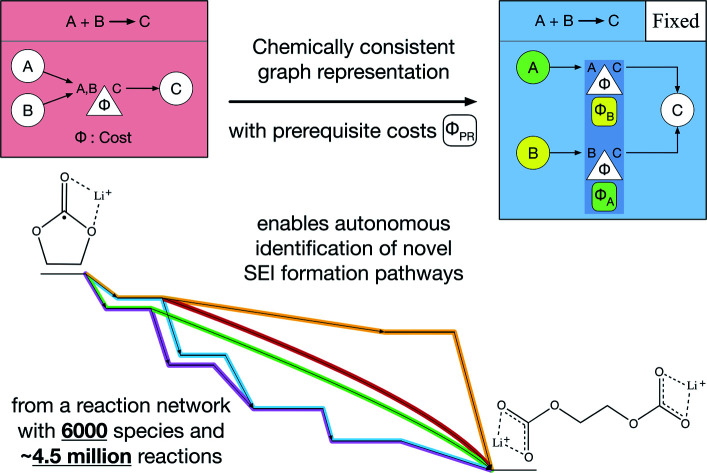

## 1. Introduction

Understanding and controlling complex reactive processes is a fundamental challenge in the development of novel chemical technologies. While computational chemistry has provided crucial insight for a broad array of reactive systems, important areas of electrochemistry,^1,2^ atmospheric chemistry,^3,4^ and metabolic biochemistry^5–7^ remain poorly understood due to their scale (in terms of number of species and reactions between them) and accompanying complexity. Moreover, critical properties and dynamics of such systems may only emerge on long timescales, particularly if they rely on key rare events, for instance reactions with high kinetic barriers. Typical atomistic modeling approaches like classical molecular dynamics (MD) and *ab initio* molecular dynamics (AIMD) are thus inherently insufficient, as they are too costly to adequately sample these rare events. On the other hand, chemical reaction networks, which use graph theory to define relationships between molecules and reactions, are well positioned to capture such complex long-time reactivity.

Reaction networks have the capacity to interrogate competing reaction mechanisms in complex chemical systems on long timescales. By simplifying chemical space into a set of connected molecule nodes and reaction nodes,^8^ reaction networks abstract away the spatial interactions of three dimensional molecular structures while preserving a realistic description of the underlying interconnected chemical mechanisms. Reaction networks can be used to compare viable reaction pathways that transform a set of starting molecules into final products or to identify optimal pathways using pathfinding algorithms.^9^ The cost to traverse a given reaction can capture the relevant thermodynamics and/or kinetics, ensuring that the “shortest” pathway (the pathway with the lowest cost) is also the one most likely to occur. Such pathway analysis inherently accounts for long-time behavior without requiring an arduously long propagation as long as important rare reactions are present in the network. Substantial work has been conducted to apply reaction networks to a wide range of scientific applications, including organic chemistry,^9–12^ retrosynthesis,^13–15^ combustion,^16–18^ catalysis,^19–21^ and sugar formation,^22,23^ as well as metabolic^7,24^ and prebiotic chemistry.^23^ However, network scale has remained fairly small; no reaction network reported in the literature built from first principles has included more than 1000 species or more than 10 000 reactions.^8^

The size of networks reported thus far has been limited in part due to the use of graph representations that cannot capture the chemical reality of multi-reactant reactions (*e.g.* A + B → C). Such representations prevent the use of optimized pathfinding algorithms. Instead, researchers employ custom approaches that can calculate path costs while respecting reaction stoichiometry.^25^ These algorithms suffer from performance and scaling limitations^11,25^ that have historically made it necessary to significantly restrict network size. As a result, reaction networks have not previously been applied to complex electrochemical or photochemical systems which may include thousands of species and millions of reactions.

The development of reaction networks to study electrochemical processes is both highly desirable and very challenging due to the extreme inherent complexity of such systems. For example, the exposure of an electrolyte to highly oxidative as well as reductive electrode materials necessitates the consideration of redox reactions coupled with irreversible chemical decomposition in a rapidly evolving local environment. These reactions form ions, fragments, and radicals, all of which may be more stable in solvent than they would be in the gas phase or in vacuum. Such factors mean that a much wider range of stable and metastable species must be included in the network than would be otherwise. As a further complication, ions and radicals are often much more reactive than neutral, closed-shell organic molecules,^26^ and their reaction mechanisms are not as well understood,^27^ precluding heuristic pruning and necessitating consideration of a vast number of reactions for each specie.

Because a reaction network capable of describing electrochemical processes must include massive quantities of species and reactions, the use of optimized pathfinding algorithms is essential. Thus, we need a graph representation that supports general network construction, including any reaction stoichiometry in a chemically consistent manner. Such an approach will ensure that all participating reaction pathways in general reaction networks of even massive size can be tractably identified.

In this article, we will first identify underlying problems in existing reaction network architectures that can potentially produce unphysical results and that prevent the use of optimized pathfinding algorithms. We then resolve this issue through the adoption of both a novel graph representation for reactions involving multiple reactant species and an iterative algorithm for the calculation of such reaction costs. To demonstrate the power of our chemically consistent graph architecture, we apply it to a network describing the electrochemical reaction cascade that forms the lithium-ion solid electrolyte interphase (SEI), a nanoscale layer that is largely responsible for battery health and capacity retention. Our approach facilitates analysis of the resulting massive reaction network containing nearly 6000 species and 4.5 million reactions, and we identify both previously accepted pathways and novel pathways to a key SEI component.

## 2. Current state of chemical reaction network graph theory

**Fig. 1:**
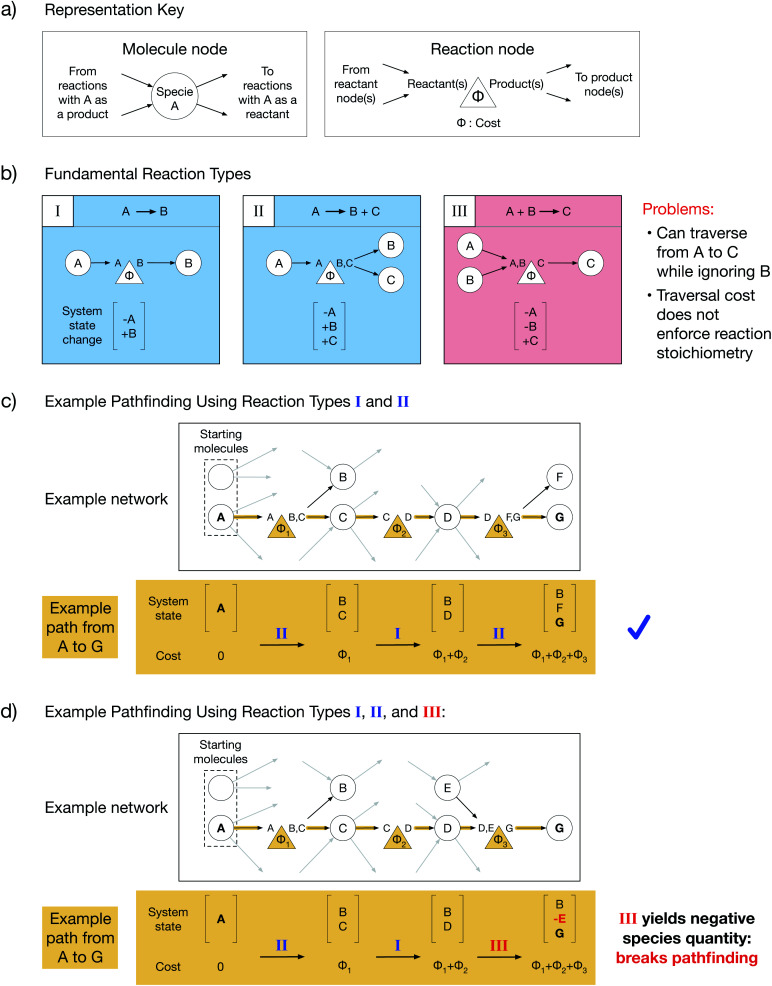
Standard reaction graph representation and multi-reactant pathfinding problem. (a) General reaction network reaction and molecule node representations, where molecule nodes are only connected to reaction nodes and *vice versa*, nodes are connected *via* directed edges according to reaction directionality, and reaction nodes have a cost, *Φ*, which must be paid in order to be traversed. (b) The three fundamental reaction types: type I – A reacts to B, type II – A reacts to B plus C, type III – A plus B react to C, where type I and type II are chemically consistent while type III is chemically inconsistent since it fails to enforce reaction stoichiometry. (c) Example network composed of only type I and type II reactions exhibits a chemically consistent path from A to G with only positive species amounts. (d) Example network including a type III reaction exhibits a chemically inconsistent path from A to G in which a negative specie occurs in the system state, demonstrating that chemically inconsistent multi-reactant reaction representations cause standard pathfinding algorithms to yield unphysical results.

Chemical reaction networks (CRNs) are graph-based data structures encoding the reactivity of a collection of molecular species using nodes and edges^8^ which can identify reaction pathways from starting molecules to desired products and facilitate comparisons between those pathways. In reality, complex reactive chemistries can explore an infinite space of possible system states. CRNs are powerful because they reduce the traversal through an unbounded space to a tractable pathfinding problem on a finite graph by ignoring system history and treating each reaction as an independent process. However, the choice of graph representations for reactions in a CRN is non-trivial. Throughout this work, we will depict molecule nodes as circles and reaction nodes as triangles (Fig. 1a). Molecule nodes will only connect to reaction nodes, and *vice versa*. Our edges encode reaction directionality: an edge directed into a molecule node must originate from a reaction node which yields that molecule as a product, and an edge directed out of a molecule node must terminate at a reaction node which consumes that molecule as a reactant (Fig. 1a). Additionally, each reaction node has an associated cost, *Φ*, which must be paid in order to traverse from reactant(s) to product(s). The cost could be a function of reaction thermodynamics, kinetics, or an experimentally derived value such as reaction yield. Summing the reaction costs along a reaction path thus yields the total cost of performing such a sequence of reactions.

We will start by considering the graph representations of three fundamental reaction types with different stoichiometries (Fig. 1b). Type I reactions are of the form A → B; type II reactions are of the form A → B + C; and type III reactions are of the form A + B → C. Each reaction implicitly defines a change in the state of the reacting system that can be addressed by simple accounting, where a system's state is specified by the amount of each species present. As a result of a reaction, the reactants must be subtracted from the state, and the products must be added to the state. Summing the initial system state and the system state changes along a reaction path yields the final system state after the reactions in the path have taken place. We note that for CRNs, which do not consider the history of the system, the system state can only be determined by *post hoc* reconstruction.

A chemically intuitive graph representation of a reaction with multiple reactants (*e.g.* type III) fundamentally conflicts with an underlying assumption of graph theory. Graph theory assumes that cost alone governs connected traversal; if node A is connected to node C, then it is always possible to traverse the graph from A to C, provided the cost is paid. Meanwhile, chemically, a reaction must obey its stoichiometry (*i.e.* all reactants must be present for a reaction to occur), and there must be a non-negative amount of each species in the system. A system state with a negative quantity of a species is thus chemically inconsistent. In a type III reaction, connectivity allows traversal from one reactant node to the product node while ignoring the second reactant, contradicting chemical laws. Such contradiction can cause chemically inconsistent reaction paths to be selected during conventional pathfinding.

When only single-reactant reactions like type I and type II are used to construct a model network, an example path from node A to node G proceeds without issue (Fig. 1c). In contrast, when a type III reaction is included in the model network, an example path from node A to node G yields a negative quantity of species E in the final system state, violating chemical assumptions and making the path chemically inconsistent (Fig. 1d). This is a fundamental problem with any current multi-reactant reaction representation. To resolve this problem, it is necessary to know the cost to create the other participating reactant during pathfinding.

Current multi-reactant representations significantly limit the utility of CRNs. Reaction networks are commonly used to identify the “best” or lowest-cost reaction paths from a set of starting molecules to any target molecule(s) of interest. The ideal tools for this task are efficient pathfinding algorithms such as Dijkstra's algorithm^28^ for single-shortest paths and Yen's algorithm^29^ for the N-shortest paths. However, as described above, these algorithms will not necessarily produce chemically valid pathways in networks containing multi-reactant reactions. It has historically been necessary to use custom methods that have suboptimal performance and scale poorly with network size based on tree traversal algorithms,^22^ breadth-first-search,^25^ or depth-first-search.^11,25^ Performant stochastic sampling approaches can instead be employed,^30^ but they are not guaranteed to find the true best solution(s). While these custom approaches may be sufficient for relatively small and simple networks, analyzing larger networks describing more complex systems will necessarily require the optimal performance of Dijkstra's and Yen's algorithms. A multi-reactant graph representation that respects both graph theoretical and chemical principles is clearly needed.

## 3. Novel graph architecture resolves multi-reactant inconsistency and enables optimized pathfinding

**Fig. 2:**
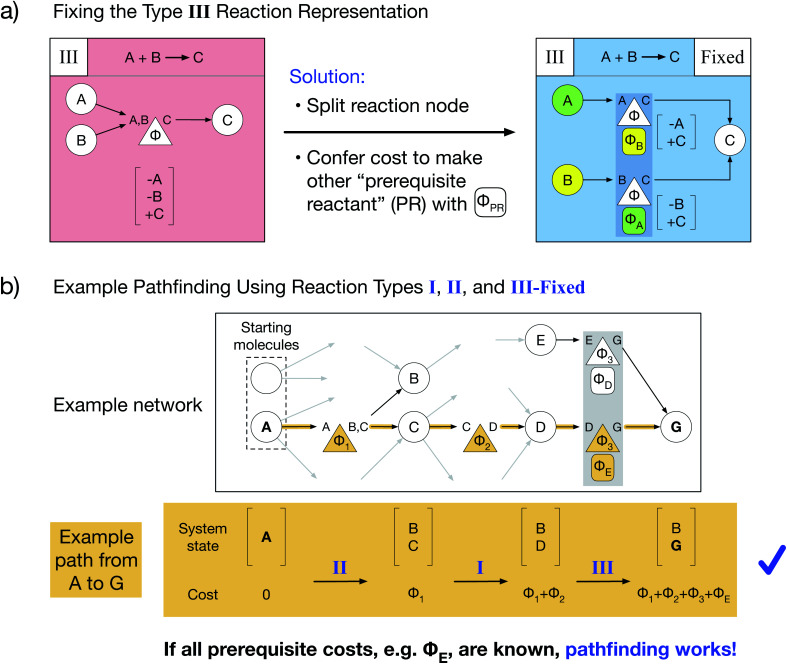
Novel type III graph representation resolves multi-reactant inconsistency and enables standard pathfinding. (a) A novel fixed multi-reactant type III reaction representation is obtained by splitting the reaction node in two and conferring the additional cost on each to make the other unconnected prerequisite reactant (PR) directly from starting molecules, denoted with the oval *Φ*_PR_ below the reaction node. (b) With the same example network as in (d), but with our fixed type III representation replacing the standard chemically inconsistent type III representation, the path from A to G no longer incurs a negative specie but instead requires paying the cost to make prerequisite E from starting molecules (*Φ*_E_). Thus, if all prerequisite costs are obtained beforehand, standard pathfinding algorithms can be used with our reaction network representation.

Here, we present a novel multi-reactant reaction graph representation that resolves previous inconsistencies by incorporating the cost to make prerequisite reactants (Fig. 2a). We split the A + B → C reaction node into two nodes which both represent the same original reaction, one node connecting A and C and the other connecting B and C. Each reaction node's cost includes both the original reaction cost *Φ* (based on the A + B → C reaction thermodynamics/kinetics or other cost function) and the additional cost to create the other “prerequisite” reactant (PR) from the available starting molecules, given by *Φ*_PR_. For example, traversing the A → C reaction node, for which B is a PR, costs *Φ* + *Φ*_B_. The PR is implicitly created by paying the PR cost and is then immediately consumed in the reaction; thus, the PR does not appear in the reaction node system state change. As a result of this transformed representation, true edge connectivity and node cost now accurately captures the chemical reality that both reactants are required for the A + B → C reaction to proceed.

Our type III representation enables the use of standard pathfinding algorithms. Consider the previous pathfinding example in which a type III reaction yielded a negative species quantity in the system state. Employing our fixed type III representation, the same example path proceeds without issue (Fig. 2b). There are two critical changes: the final system state no longer includes a negative quantity of species E, and the total path cost additionally includes the cost of producing prerequisite E (*Φ*_E_) as part of the last reaction. Thus, our novel type III representation resolves the multi-reactant reaction inconsistency and enables pathfinding *via* optimized Dijkstra's and Yen's algorithms for any chemical reaction network. However, our representation additionally requires that prerequisite costs be solved and included in reaction node costs before pathfinding is performed.

**Fig. 3:**
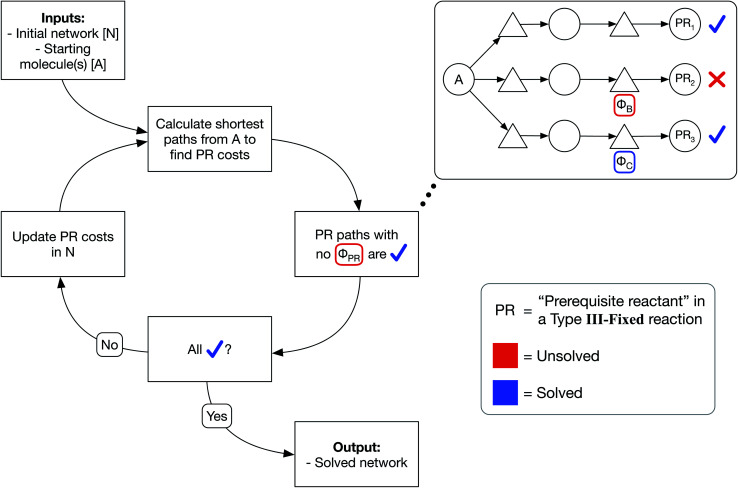
Iterative prerequisite cost solving algorithm. Starting with an initial network N and starting molecules A, we find the shortest path from A to each specie in N. We then identify solved prerequisites where the shortest path to a given specie node itself contains no unsolved prerequisites. If unsolved prerequisites remain, all prerequisite costs are updated according to the solve shortest paths, and another iteration is performed. Eventually, all prerequisites will be solved and the final network will be returned.

We have developed an iterative algorithm to simultaneously solve all prerequisite costs before pathfinding. Naively attempting to solve PR costs on the fly often results in unbound recursion. Instead, we iteratively solve all PR costs with the following procedure outlined in Fig. 3:

(1) We calculate the shortest path from each starting molecule to every possible molecule in the network that may act as a prerequisite using Dijkstra's algorithm.

(2) We then identify solved PRs in which the shortest path to that molecule node itself has no unsolved PRs.

(3) If any PRs remain unsolved, we update all PR costs in the network and continue iterating until all PRs are solved.

In this manner, PR costs at any iteration are an effective lower bound on the true PR costs. The cost of each unsolved PR will rise from one iteration to the next until that PR is solved, at which point the cost remains fixed through all further iterations. Additionally, by only updating the network at the end of each iteration, PR costs are uniquely defined and do not depend on the implementation details of Dijkstra's algorithm such as the order that the nodes are searched. We show an example of PR solving for a model network in Section S1 of the ESI.† While this example and the concept of PR solving are quite simple, we note that the PR solving process is the key to allowing optimized pathfinding in reaction networks and is the most computationally intensive step of our chemically consistent graph architecture.

One subtlety of PR costs is the temptation to think that a species created incidentally as a byproduct of a type II reaction (for example, molecule B in Fig. 2b) could then be used as a prerequisite for a later reaction, thus avoiding the need to pay *Φ*_PR_ and reducing the cost of the path overall. However, a CRN-based approach cannot accommodate such a cost reduction since system history is ignored and reaction costs must be independent, which is further enforced by the greedy nature of Dijkstra's algorithm. This is a limitation of our methodology, since real chemical processes may contain paths where byproducts from one step are consumed as reactants multiple steps later. However, we note that in the chemical application considered in this work, this synergy is not relevant to the paths identified.

## 4. Autonomous identification of optimal reaction pathways in SEI formation

Here we demonstrate the application of our chemically consistent graph architecture to the formation of a particular component of the Li-ion battery solid-electrolyte interphase. When the electrolyte reductive stability limit is reached during the initial charging of a Li-ion battery, a cascade of interdependent reactions – including reduction, oxidation, bond cleavage, and bond formation – spontaneously occur to form an SEI on the battery anode surface.^31^ This process is critical to cycling performance, as the formation of a functional, passivating SEI enables many battery technologies to operate outside of the thermodynamic stability limit of the electrolyte.^32–36^ Specifically, the SEI needs to conduct metal ions while preventing electrical conduction and remaining chemically stable to minimize undesirable ongoing electrolyte or anode degradation.^37^

Understanding the mechanisms of SEI formation is critical to the development of battery technologies. Such understanding could allow researchers to modify the initial battery conditions or electrolyte composition to suppress or promote targeted reaction pathways, thereby engineering and controlling the properties and composition of the SEI. With the capability to predict the formation of SEI components and the properties of the resultant SEI based solely on the initial conditions of the electrolyte, it would be possible to computationally screen the vast chemical space of potential electrolyte salts, solvents, and additives to guide experimental investigations and expedite the development of novel next-generation battery chemistries beyond lithium-ion.

Over roughly two decades of dedicated research into SEI formation, it has become accepted that lithium ethylene dicarbonate, also known as Li_2_EDC or LEDC, is a major, early-formed organic component of the interphase in Li-ion batteries, based on both experimental and computational results.^38–41^ A number of pathways from the ethylene carbonate (EC) solvent molecule and Li^+^ to LEDC have been proposed in the literature. From these, two prominent paths have emerged. In the “one-electron” path, two reduced EC molecules, coordinated with lithium (Li^+^EC^−^), ring-open and combine to form LEDC and ethylene.^39^ In the “two-electron” path, after ring-opening, one LiEC molecule is reduced again and decomposes to form ethylene and LiCO_3_^−^. This LiCO_3_^−^ then attacks an EC molecule to form LiEDC^−^, which then coordinates with a Li^+^ to form LEDC.^38,42,43^ Here, we employ a reaction network to determine if these proposed pathways are indeed the most thermodynamically competitive, or if there are other pathways that may contribute to LEDC formation.

Our reaction network methodology, based on a novel graph representation for multi-reactant type III reactions and an iterative PR cost solving algorithm, is uniquely suited to provide insights into SEI formation processes. Because the SEI forms spontaneously on an electrified interface in the presence of multiple species and local environments, there is a vast number of possible reactive fragments which in turn gives rise to millions of plausible reactions. As a result, a reaction network describing SEI formation will necessarily be of massive scale to ensure that key pathways are not missed, making such a network an ideal test case for our approach. We emphasize that attempting to analyze such a reaction network with millions of reactions is completely unprecedented and is only feasible with optimized pathfinding algorithms.

We have constructed a reaction network to describe SEI formation using our chemically consistent graph architecture that contains 6000 species and nearly 4.5 million reactions. We employ a thermodynamic cost function *Φ* = *C* + *e*^Δ*G*/*kT*^, where Δ*G* is the free energy of the reaction and *C* is a constant that provides a lower bound on reaction cost and encourages shorter paths. We use *C* = 1 in this work based on empirical investigation and the physically reasonable result that, with this choice, one reaction with Δ*G* = 0 costs an equivalent amount as two reactions with Δ*G* ≪ 0. Further discussion of the cost function and our procedure for network construction is presented in Section S2 of the ESI.†

**Fig. 4:**
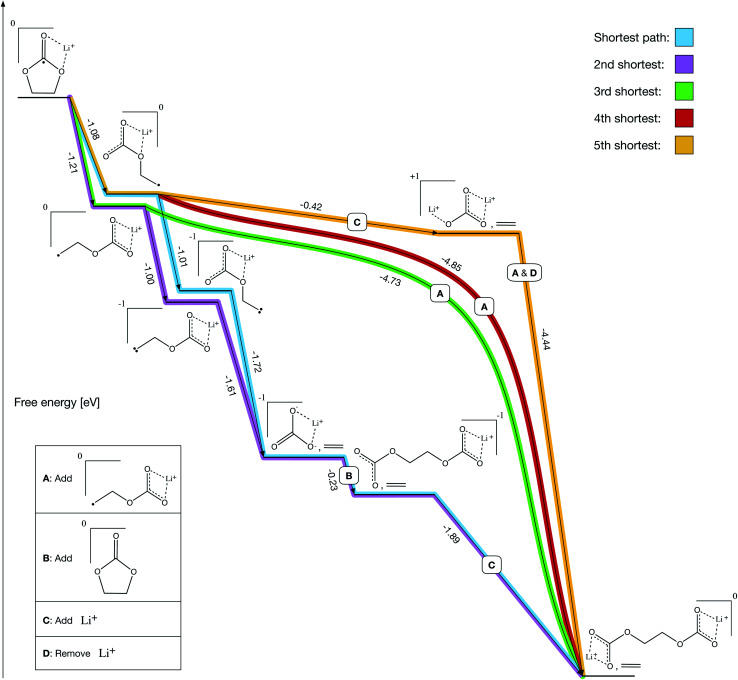
LEDC five shortest paths. The 2nd shortest path (purple) and the 3rd shortest path (green) were the two mechanisms previously proposed in the literature and each contain one two-bond reaction. The shortest path (blue) and 4th shortest path (red) follow very similar mechanisms to purple and green, respectively, but they contain a counterintuitive ring-opening step (−1.08 eV) which has not been previously considered. Finally, the 5th shortest (gold) path exhibits both the counterintuitive ring-opening and stabilization *via* a transient Li-ion that has never been previously proposed.

After solving the prerequisites of our final network, pathfinding autonomously identifies both previously reported and novel formation mechanisms for LEDC (Fig. 4). We define Li^+^ and ethylene carbonate (EC) as our starting molecules and are able to solve all prerequisite costs in 19 iterations over the course of 14 hours on a laptop. Prior to the reactions shown in Fig. 4, all paths include the bidentate coordination of Li^+^ with EC followed by reduction.

A key validation of our approach is that both previous prominent mechanisms, proposed through manual investigations – the two-electron path (Fig. 4, purple) and the one-electron path (Fig. 4, green) – are recovered as our 2nd shortest and 3rd shortest paths, respectively. The shortest and 4th shortest paths (Fig. 4, blue and red) are nearly equivalent to the 2nd and 3rd shortest paths, respectively, with the blue path being another two-electron mechanism and the red path being another one-electron mechanism. However, both include a counterintuitive ring-opening step that has not been previously considered because it is slightly less thermodynamically favorable than the conventional ring-opening mechanism (−1.08 eV *vs.* −1.21 eV). Intuitively, a chemist would select the more favorable route at a given step, but in this case non-intuitive reactions could meaningfully contribute.

The 5th shortest path (Fig. 4, gold) is entirely novel and leverages a transient lithium ion to decompose ring-opened Li^+^EC^−^ exergonically. Note that decomposing ring-opened Li^+^EC^−^ in isolation is slightly endergonic according to our calculations (+0.25 eV), which may be why it has not previously been reported. However, our procedure autonomously identified that the coordination of an additional lithium ion simultaneous with the decomposition yields a much more thermodynamically favorable reaction (−0.42 eV), perhaps making it competitive with the other mechanisms emerging from the network. The additional lithium ion can then dissociate simultaneously with the addition of a ring-opened Li^+^EC^−^ to form LEDC (−4.44 eV), making it a transient participant in the gold pathway.

Since our network contains only thermodynamic information, reaction costs are a function of free energy changes. While a more accurate cost would be based on reaction kinetics, calculating reaction barriers is extremely computationally intensive and challenging to automate. In the case of LEDC formation, the electron transfer rate has further been shown to be important in determining pathway fitness;^44^ in principle, electron transfer should also be accounted for. While we currently do not have any kinetic information in our network, including such information would not require any modification to the reaction representation or pathfinding algorithm.

The ability to identify the best reaction paths in highly complex reaction networks is the first step towards reverse engineering SEI composition and complex reactivity more generally. The best paths identified by our approach are the natural targets for reaction engineering. Paths that yield desirable products provide a set of reactions that can be strategically promoted, through additives and concentration tuning, while paths that yield undesirable products instead identify reactions that may be selectively hindered. Such reaction engineering leveraging knowledge of desirable and undesirable reactions has already been put into practice in the field of heterogeneous catalysis,^45–48^ where substrates are chosen to minimize parasitic reactions and maximize output of the desired chemical.

## 5. Conclusion

We have developed a novel reaction network architecture that accommodates any reaction stoichiometry. We split reactions with multiple reactants into multiple reaction nodes that each include the cost of creating the other prerequisite reactant. We have additionally developed a procedure to iteratively solve for all prerequisite costs. Our approach allows optimized shortest path algorithms like Dijkstra's and Yen's to be used to identify the best or N-best reaction paths to any given molecule node in any chemical reaction network for the first time. The ability to use these algorithms facilitates pathway analysis on much larger chemical reaction networks, which can describe much more complex systems, than previously possible.

Using our chemically consistent architecture, we have generated a thermodynamic network that contains nearly 6000 species and roughly 4.5 million reactions, the largest chemical reaction network ever built from first principles. Pathfinding on the resulting network to the early-SEI component LEDC identifies both previously-proposed mechanisms and three new possible mechanisms, all in an automated fashion. Identification of optimal paths informs which reactions could be promoted or hindered to control the abundance of beneficial or detrimental species, a key step towards the goal of engineering SEI composition and controlling complex reactive systems in general.

Future work will include pathfinding to other important SEI components and investigating the impact of additives on SEI formation pathways. We are also developing an automated procedure to calculate and incorporate reaction barriers into the network as well as an automated approach to adding reactions with more than two bonds changing or with one or more bonds changing simultaneously during redox processes. With this highly general, robust, and scalable approach, it will be possible to understand and control complex chemical or electrochemical reactive systems, expediting the development of novel materials and chemical technologies in domains such as energy storage and medicine and improving upon synthetic pathways for industrial applications.

## Conflicts of interest

There are no conflicts to declare.

## Supplementary Material

SC-012-D0SC05647B-s001
