# Supplementary material for: A chemically consistent graph architecture for massive reaction networks applied to solid-electrolyte interphase formation
Source: Chem Sci. 2021 Feb 24;12(13):4931–9. doi: 10.1039/d0sc05647b (PMC8179555; doi:10.1039/d0sc05647b)
Supplement: SC-012-D0SC05647B-s001 [file SC-012-D0SC05647B-s001.pdf]

Electronic Supplementary Material (ESI) for Chemical Science.
This journal is © The Royal Society of Chemistry 2021

A chemically consistent graph architecture for massive reaction networks applied to solid-electrolyte interphase formation: Supporting Information

Samuel M. Blau, Hetal D. Patel, Evan Walter Clark Spotte-Smith, Xiaowei Xie, Shyam Dwaraknath, and Kristin A. Persson*

E-mail: kapersson@lbl.gov

S1: Simple Prerequisite Solving Example

Figure S1 shows the prerequisite costs of a simple network being solved in two iterations. The network contains nine molecules (A, B, D, E, F, H, L, Y, and Z) and ten reactions:

1. A $\rightarrow$ B, $\phi = 6$
2. A $\rightarrow$ D + E, $\phi = 1$
3. A $\rightarrow$ Z + Y, $\phi = 2$
4. A $\rightarrow$ F, $\phi = 4$
5. Z $\rightarrow$ B, $\phi = 3$
6. D + B $\rightarrow$ L, $\phi = 1$
7. E + Z $\rightarrow$ F, $\phi = 2$

8. Y + Y → L, $\phi = 4$

9. B + B → H, $\phi = 3$

10. L + F → H, $\phi = 1$

Reactions 6-10 are all Type III, and thus involve prerequisite costs. Note that for reactions 8 and 9, both reactants are the same species. Thus, these two reactions can each be represented with one reaction node with the relevant prerequisite cost instead of two.

We define specie A as our starting molecule and specie H as our target molecule. Our goal is to find the shortest path from A to H, along with its associated cost. We must first solve all necessary prerequisite costs. Our network begins with all PR costs set to zero (Fig. S1 I). We then check the shortest path from A to H to determine if unsolved PR costs remain. The highlighted shortest path has a cost of three but does indeed contain unsolved PR costs for species B and F, as indicated by the red outlines of the PR costs. We determine all minimum PR costs by finding the shortest paths from A to each other species in the network (Fig. S1 II). We identify that the shortest paths to species D, E, Z, Y, and B contain no unsolved PRs, while the shortest paths to species L and F do contain unsolved PRs - B and Z, respectively. At this point, L, F, and H remain unsolved, as denoted by the red X, while the other five species are solved, as denoted by the blue checks.

Following the first iteration of PR solving, we update the PR costs in our network (Fig. S1 III) and repeat the procedure. The new highlighted shortest path from A to H takes a different route through the network due to the updated PR costs. It has a cost of seven and contains one unsolved PR cost for species L. We then identify the shortest paths from A to L and from A to F, which remained unsolved after the previous iteration. We find new minimum costs of seven and four, respectively (Fig. S1 IV), along paths that contain no unsolved PRs. Thus, L and F are now also solved, as denoted by the blue checks. We then update these two PR costs in our network (Fig. S1 V) and find the new shortest path from A to H has a minimum cost of 12. This path contains no unsolved PRs (Fig. S1 VI), so we have achieved our goal.

While the three highlighted shortest paths from A to H obtained at different iterations of PR solving initially appear distinct, they are actually all the same path and all have a cost of 12 once PRs are solved. This is not always the case, but in this example, all three highlighted

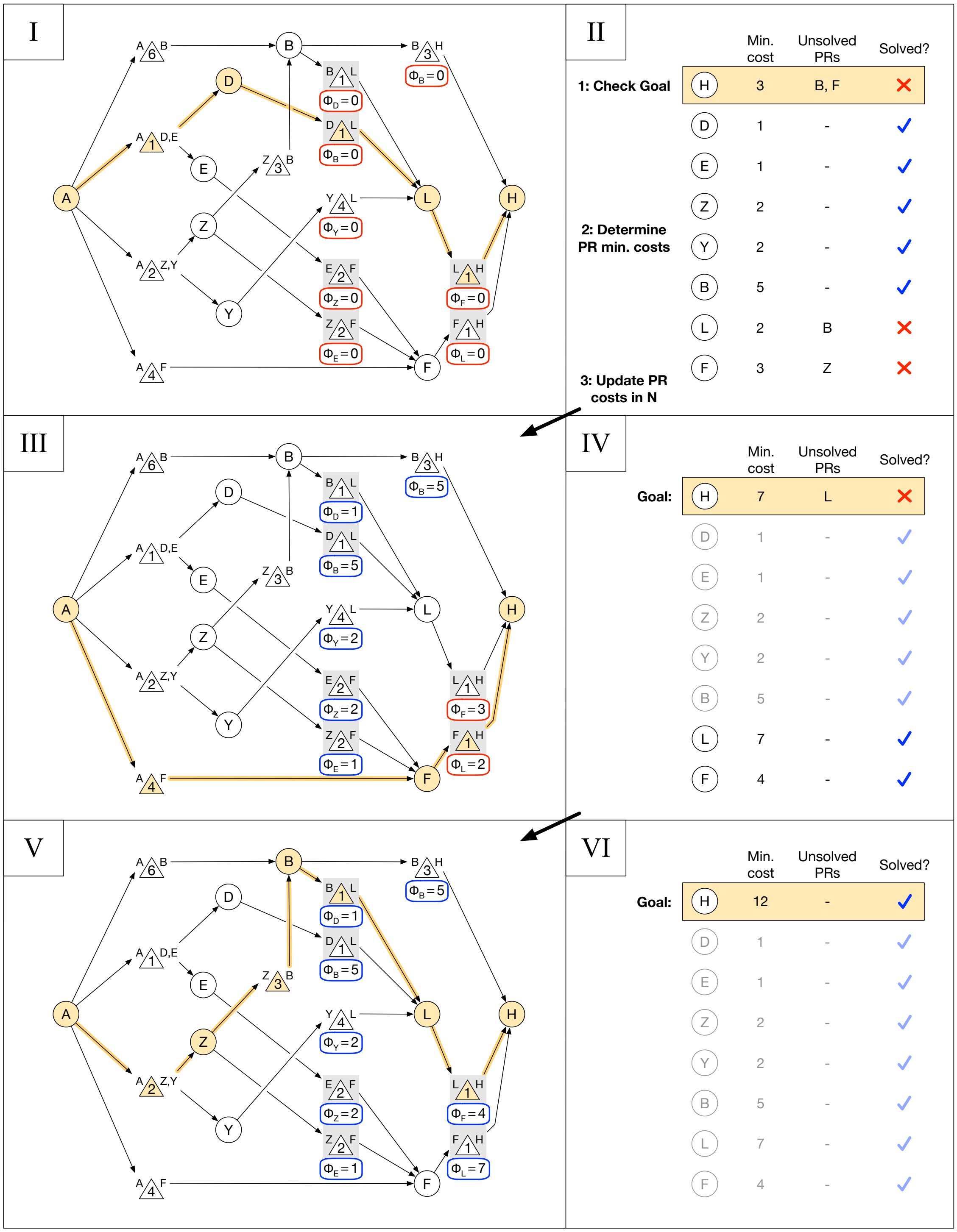

Figure S1: **Simple prerequisite cost solving example.** **I**. Initial network with all PR costs set to zero where the initial shortest path from our reagant, A, to our target, H, is highlighted. **II**. One iteration of the PR solving procedure. **III**. Network with updated PR costs following the first iteration. **IV**. Second cost update iteration. **V**. Network with updated PR costs following the second iteration. **VI**. Solved total cost for the shortest path from A to H.

paths fundamentally describe the same sequence of reactions. The highlighted path from A to H in Fig. S1 V includes PR costs for species D and F as part of reactions 6 and 10, respectively. Thus, in addition to this "main path", we are also implicitly traversing from A to D, as in the highlighted path of Fig. S1 I, such that the main path can move from B to L via reaction 6. We are also implicitly traversing from A to F, as in the highlighted path of in Fig. S1 III, such that the main path can move from L to H via reaction 10. These implicit supplementary traversals are captured by the prerequisite costs. The optimal series of reactions that yield H from a reservoir of starting molecule A are entirely identical if the main path goes through species node B and pays the prerequisite cost of D or if the main path goes through species node D and pays the prerequisite cost of B. Either way, reaction 6 is included in the reaction sequence. The fact that all paths which describe the same sequence of reactions have the same cost after PR solving is precisely the chemical consistency that our graph architecture was developed to enforce.

S2: Reaction Network Construction and the Cost Function

In order to construct a reaction network with minimal imposed bias, we simulated all unique subfragments of all principle molecules posited to be potentially relevant to lithium-ion ethylene carbonate (EC) SEI formation at charges of -1, 0, and +1, yielding 5,979 unique species. Initial principle molecule 3D structures were constructed by-hand and then optimized to potential energy surface minima at charges of 0, -1, and +1. Small-scale conformer analysis was also performed by-hand to ensure that principle molecule geometries were in a plausible low-energy conformation. Initial fragment geometries were then constructed by enumerating all isomorphically unique connected molecular subgraphs of all optimized principle molecule geometries, where a molecule is represented as a graph with atoms as nodes and bonds as edges. Each initial fragment geometry corresponds to a unique molecular graph, and thus no two initial fragments exhibit equivalent bonding. All initial fragment geometries were then optimized to potential energy surface minima at charges of 0, -1, and +1. However, while we did not explicitly perform a conformer analysis for

fragments, many fragment structures changed bonding during optimization, often yielding multiple isomorphic structures. By only including the structure with the lowest free energy in such cases, we have obtained some of the benefits of a conformational analysis without imposing additional computational cost.

We obtained optimized geometries and free energies of each specie using Q-Chem 5.2.2,[1] the ωB97X-V density functional,[2] the def2-TZVPPD basis set,[3,4] and the SMD implicit solvent model,[5] defining a solvent with a dielectric constant of 18.5 (the dielectric constant of a 3:7 ethylene carbonate-ethyl methyl carbonate solution)[6] and all EC short-range parameters.[7] Such a high level of theory was necessary to accurately capture the charged, open-shell, metal-coordinated, solvated species that make up the vast majority of our network. A reliable description of bonding was obtained by analyzing the electron density critical points with the Critic2 software.[8] We built an advanced workflow infrastructure including automated on-the-fly error correction to ensures that all geometries are at true potential energy surface minima and to obtain results in a reasonable amount of time.[9] Overall, the calculations for the preliminary SEI formation reaction network described here cost over 30 million CPU-hours across both the National Energy Research Supercomputing Center's Cori supercomputer and the National Renewable Energy Laboratory's Eagle supercomputer.

With our unique species in hand, we identified the following basic reactions from the bottom up by comparing species molecular charge and bonding:

- One-electron oxidation/reduction with no change in bonding (Type I, 4434 reactions)
- Intramolecular one-bond breakage/formation (Type I, 1904 reactions)
- Intermolecular/coordination bond breakage (Type II, 54854 reactions)
- Intermolecular/coordination bond formation (Type III, 54854 reactions)

Our approach identified over 116,000 reactions, approximately an order of magnitude more than any reaction network reported in literature, though without any kinetic barriers at this point. We set the electron free energy to be -1.897 eV such that the monodentate reduction of Li^+EC has a free energy change of zero, signifying a reductive tipping point. This was a somewhat arbitrary choice, and it does not impact the relative rankings of the paths we obtain.

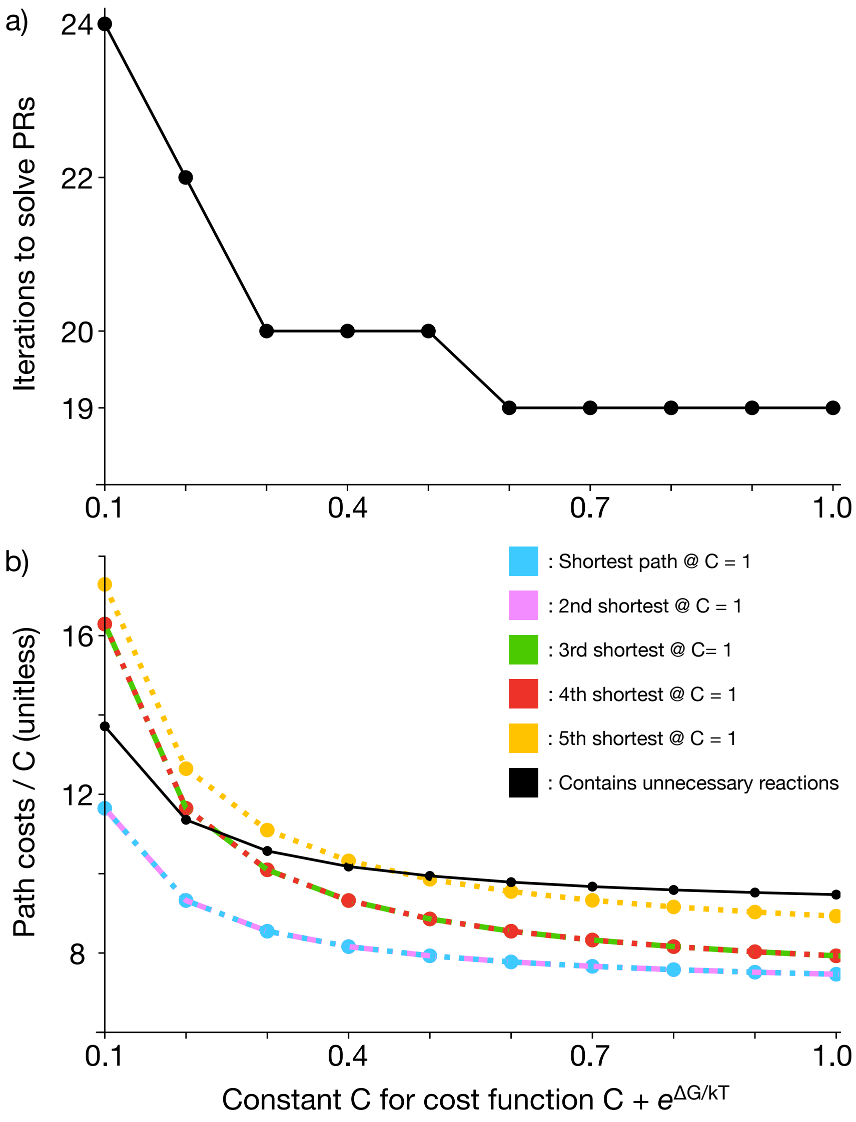

Figure S2: **The impact of the constant factor C on the cost function** $\Phi = C + e^{\Delta G/kT}$**.** **a**. Number of iterations required to solve prerequisites as C increases from 0.1 to 1.0. **b**. Relative path costs (path costs divided by C) as C increases from 0.1 to 1.0 for the five shortest paths shown in Figure 4 (which used $C = 1$) and another path (black) that is entirely equivalent to the shortest path but with two additional unnecessary $\Delta G < 0$ reactions.

In order to obtain an effective cost from a given reaction free energy change ΔG, we sought to employ a physically-motivated function that satisfied the following three considerations:

- The cost function should be continuous to avoid discontinuities that could produce unphysical behavior (for instance, where reactions with $\Delta G = +0.001$ eV and $\Delta G = -0.001$ eV have vastly different costs despite nearly identical ΔG values).

- $\Delta G < 0$ reactions should not have such small costs that it becomes facile to add unnecessary reactions (based on the principle that a simpler reaction path with fewer steps is more likely than a more complex reaction path with more steps, all other things being equal).
- The prerequisite solving procedure should reach convergence as quickly as reasonably possible.

We use the cost function $\Phi = C + e^{\Delta G/kT}$, where C is a constant unitless value that empirically captures the base cost of performing any reaction independent of its thermodynamics or kinetics. C also serves as a lower bound on reaction cost, ensuring that the prerequisite solving procedure can iterate towards convergence and encouraging shorter paths overall.

In order to determine the value of C to use, we investigated values from 0.1 to 5.0. Figure S2 shows how the number of iterations required to solve the PRs (Figure S2a) and the relative costs of the shortest paths (Figure S2b) change as a function of C. We only plot up to $C = 1.0$ because the number of PR solving iterations and the path orderings remain unchanged for larger values of C. Figure S2b plots relative path cost (path cost divided by C) to more clearly show path ordering in the absence of the linear increase in absolute cost with increasing C. In addition to the five paths discussed in the main text, Figure S2b also includes a path, shown in black, which is entirely equivalent to the shortest path but with two additional unnecessary $\Delta G < 0$ reactions that together yield Li^+ from starting molecules (despite Li^+ itself being a starting molecule).

With our choice of C, we seek to ensure that $\Delta G < 0$ reactions are not so low in cost that they can be added onto other paths to create meaningless duplicates, complicating shortest path searches. Our investigation revealed that $C \geq 0.5$ yields paths in order of their number of exergonic ($\Delta G < 0$) steps (7 - blue, purple, red, and green; 8 - gold; 9 - black). Further, the number of iterations required to solve prerequisites decreases as C increases until stabilizing at 19 iterations for $C \geq 0.6$. Thus, for the network presented in this work, the specific choice of C at or above 0.6 appears to be essentially arbitrary.

We have chosen to use $C = 1$ for our standard cost function, equivalent to the value of the exponential factor for a $\Delta G = 0$ reaction. This choice also enforces that one $\Delta G = 0$ reaction costs an equivalent amount as two $\Delta G << 0$ reactions, which seems physically reasonable for a purely thermodynamic network. While we expect the functional form of the cost function to be generally

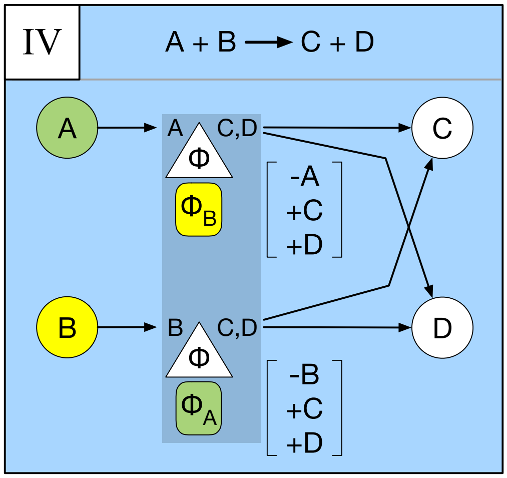

Figure S3: **Type IV reaction representation**.

transferable between networks, the specific choice of the constant C may need to be evaluated on a case-by-case basis. However, we note that once costs are based on kinetic barriers, as would be more accurate and realistic, the value of the constant will minimally impact overall costs since the exponential term will generally dominate.

Besides the four basic classes of reactions identified previously, reactions with two simultaneous bond changes have been proposed to be important for SEI formation[10–13] and thus must be included in our network to obtain realistic reaction paths. However, reactions with two bonds changing can have multiple reactants and multiple products and thus require reaction node motifs beyond Types I, II, and III introduced previously. Since we now have viable representations for multiple reactants (Type III) and multiple products (Type II), we can trivially construct a representation for any number of reactions and any number of products. The most common such reaction would be of the form A + B → C + D, which we will define as Type IV. A diagram of the Type IV graph representation is shown in Figure S3.

In order to avoid a combinatorial explosion of two-bond reactions, we have included only those reactions that are likely to have an impact on the prominent reaction paths. Specifically, we only include those two-bond reactions that avoid an endergonic step (Figure S4). By combining two reactions, the first endergonic and the second exergonic, the cost of traversing the two-bond reaction becomes substantially less than sequentially traversing the component one-bond reactions.

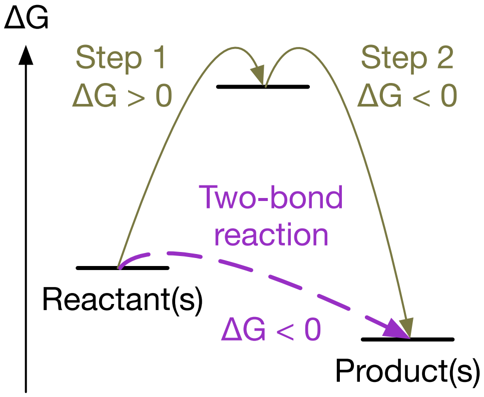

Figure S4: **Model two-bond reaction which skips an endergonic intermediate**.

We ignore two-bond reactions that are overall endergonic, as such reactions are thermodynamically unfavorable and unlikely to proceed. We also ignore two-bond reactions where a single atom other than lithium is the endergonic intermediate. Lithium is excepted both because it is a starting molecule and because it is expected to change its coordination environment in the electrolyte and in the SEI to allow for diffusion. No other single-atom "hopping" reactions have been previously proposed or reported to be relevant to any SEI formation mechanisms or reaction pathways, which we take as a justification of this exclusion. Using these criteria, considering only exergonic, non-hopping two-bond reactions which skip an endergonic intermediate, we identify 4.35 million two-bond reactions in total, with over 99.5% being Type IV.

Our final network after the addition of two-bond reactions contains 19,713 Type I reactions, 55,609 Type II reactions, 61,124 Type III reactions, and 4,333,996 Type IV reactions, for a total of 4,470,442 reactions.

Despite attempts to minimize imposed bias during network construction, our bottom-up reaction identification approach and the scope of our calculations are inherent limitations, though our representation and pathfinding approach remain general and unbiased. We cannot guarantee that every possibly relevant specie is present in our network, since we only simulated charges of -1, 0, and 1, only simulated singlet and doublet spin multiplicities, and performed very limited conformational sampling. Further, we did not account for more complex reaction mechanisms beyond two simultaneous bond changes, such as concerted dissociative redox or reactions in which

more than two bonds change simultaneously, given that billions of reactions would be identified if we included such reactions. We also have not calculated reaction transition states, which are needed to confirm a reaction's existence and to get more accurate reaction costs. However, despite the inherent challenges in constructing a massive electrochemical reaction network, we stress that our novel multi-reactant representation and iterative prerequistite solving procedure are entirely general and can allow optimized shortest path algorithms to be applied to any reaction network, thereby enabling the construction and analysis of far larger networks than ever before and thus making the analysis of far more complex systems possible.

The Q-Chem data set and Python code used to generate and analyze the reaction network will be made publicly available in the coming months to both ensure that our results are reproducible and allow our chemically consistent graph architecture to be used by the community. The publication of this manuscript is a key step towards our planned public release.

References

(1) Shao, Y. et al. Advances in molecular quantum chemistry contained in the Q-Chem 4 program package. *Mol. Phys.* **2015**, *113*, 184–215.

(2) Mardirossian, N.; Head-Gordon, M. ωB97X-V: A 10-parameter, range-separated hybrid, generalized gradient approximation density functional with nonlocal correlation, designed by a survival-of-the-fittest strategy. *Phys. Chem. Chem. Phys.* **2014**, *16*, 9904–9924.

(3) Weigend, F.; Ahlrichs, R. Balanced basis sets of split valence, triple zeta valence and quadruple zeta valence quality for H to Rn: Design and assessment of accuracy. *Phys. Chem. Chem. Phys.* **2005**, *7*, 3297.

(4) Rappoport, D.; Furche, F. Property-optimized Gaussian basis sets for molecular response calculations. *J. Chem. Phys.* **2010**, *133*, 134105.

(5) Marenich, A. V.; Cramer, C. J.; Truhlar, D. G. Universal Solvation Model Based on Solute Electron Density and on a Continuum Model of the Solvent Defined by the Bulk Dielectric Constant and Atomic Surface Tensions. *J. Phys. Chem. B* **2009**, *113*, 6378–6396.

(6) Hall, D. S.; Self, J.; Dahn, J. R. Dielectric Constants for Quantum Chemistry and Li-Ion Batteries: Solvent Blends of Ethylene Carbonate and Ethyl Methyl Carbonate. *J. Phys. Chem. C* **2015**, *119*, 22322–22330.

(7) Qu, X.; Jain, A.; Rajput, N. N.; Cheng, L.; Zhang, Y.; Ong, S. P.; Brafman, M.; Maginn, E.; Curtiss, L. A.; Persson, K. A. The Electrolyte Genome project: A big data approach in battery materials discovery. *Comput. Mater. Sci.* **2015**, *103*, 56–67.

(8) Otero-de-la Roza, A.; Johnson, E. R.; Luaña, V. Critic2: A program for real-space analysis of quantum chemical interactions in solids. *Comput. Phys. Commun.* **2014**, *185*, 1007–1018.

(9) Blau, S. M.; Spotte-Smith, E.; Wood, B.; Dwaraknath, S.; Persson, K. Accurate, Automated Density Functional Theory for Complex Molecules Using On-the-fly Error Correction. *ChemRxiv* **2020**,

(10) An, S. J.; Li, J.; Daniel, C.; Mohanty, D.; Nagpure, S.; Wood, D. L. The state of understanding of the lithium-ion-battery graphite solid electrolyte interphase (SEI) and its relationship to formation cycling. *Carbon* **2016**, *105*, 52–76.

(11) Wang, A.; Kadam, S.; Li, H.; Shi, S.; Qi, Y. Review on modeling of the anode solid electrolyte interphase (SEI) for lithium-ion batteries. *Npj Comput. Mater.* **2018**, *4*, 15.

(12) Burkhardt, S. E. Impact of Chemical Follow-up Reactions for Lithium Ion Electrolytes: Generation of Nucleophilic Species, Solid Electrolyte Interphase, and Gas Formation. *J. Electrochem. Soc.* **2017**, *164*, A684–A690.

(13) Li, T.; Balbuena, P. B. Theoretical studies of the reduction of ethylene carbonate. *Chem. Phys. Lett.* **2000**, *317*, 421–429.
